# Effect of Chlorination on the Shape Resonance Spectrum of CH_2_Br_2_ and CCl_2_Br_2_ Molecules in Collisions with Electrons

**DOI:** 10.1021/acs.jpca.4c04987

**Published:** 2024-09-26

**Authors:** Murilo O. Silva, Giseli M. Moreira, Romarly F. da Costa, Márcio H. F. Bettega

**Affiliations:** † Instituto Federal do Paraná, Campus Avançado Goioerê, Rodovia Luiz Dechiche, s/n, 87360-000 Goioerê, Paraná, Brazil; ‡ Departamento de Física, Universidade Federal do Paraná, Caixa Postal 19044, 81531-980 Curitiba, Paraná, Brazil; § Centro de Ciências Naturais e Humanas, Universidade Federal do ABC, 09210-580 Santo André, São Paulo, Brazil; ∥ Departamento de Física, Universidade Estadual do Centro-Oeste, 85040-167 Guarapuava, Paraná, Brazil

## Abstract

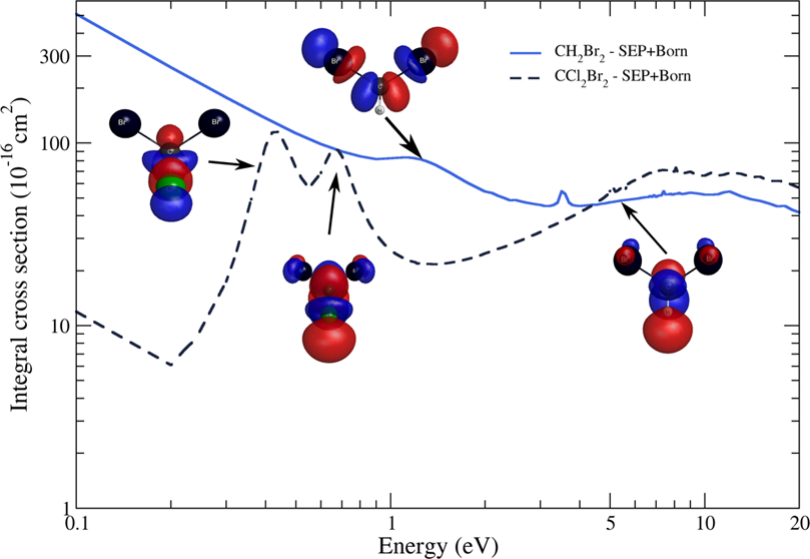

In this work, we present a theoretical study of elastic electron scattering by the CH_2_Br_2_ and CCl_2_Br_2_ molecules using the Schwinger multichannel method. Through the scattering amplitudes computed at the static-exchange and static-exchange plus polarization levels of approximation, we have obtained integral, momentum transfer, and differential cross sections for energies ranging from 0 to 20 eV. For both systems, the resonance spectrum was identified in the cross sections and characterized with basis on the analysis of the eigenvalues and eigenvectors obtained by diagonalization of the scattering Hamiltonian. Despite some discrepancies, our cross sections and resonance assignments are in overall good agreement with the experimental and theoretical data available in the literature. Present results also indicate that the effect of chlorination (i.e., the substitution of the hydrogen atoms by chlorine atoms in going from CH_2_Br_2_ to CCl_2_Br_2_) on the scattering process causes the following outcomes: the change in the position of the resonances in the low-energy regime and the increase in the magnitude of the cross sections for higher energies.

## 1 Introduction

Collision processes in planetary atmospheres play a key role in energy exchange and chemical dynamics occurring in such environments. These processes involve interactions between electrons and photons with neutral and ionized species, resulting in phenomena such as excitation, ionization, and electron attachment. Notable examples of these phenomena include auroras in the Earth’s atmosphere.^1^ In atmospheric environments, concerns grow when considering the climate change that has significantly impacted our planet. One major contributor to the Earth’s temperature balance is tropospheric ozone, which is involved in a complex network of interactions with other atmospheric gases and particles. Global levels and exposure patterns to ozone have changed dramatically over the last 50 years, impacting global warming, air quality, food production, and ecosystem function.^2^ To address this issue, the release of organic compounds containing bromine into the atmosphere has raised concerns due to their potential adverse impact on stratospheric ozone. Compounds such as CH_3_Br, CH_2_Br_2_, and other halogenated substances can be decomposed by photochemical processes and reactions with OH radicals, contributing to ozone layer depletion. Studies also indicate that chlorine atoms, resulting from the photodissociation of chloromethanes and fluorochloromethanes released into the atmosphere, participate in a chain of reactions that leads to the removal of ozone molecules.^3,4^ Understanding the removal mechanisms and environmental effects of these compounds is crucial for assessing their impact and developing mitigation strategies.^5^ To comprehend these processes, electron collision cross sections are of great importance. These cross sections, determined through experimental and theoretical investigations, provide essential data for modeling and interpreting observations of planetary atmospheres.^1^

Motivated by these considerations, we present results on elastic scattering of low-energy electrons by halomethane molecules, specifically dibromomethane (CH_2_Br_2_) and dichlorodibromomethane (CCl_2_Br_2_). The interaction between electrons and these systems has been previously studied in the literature. Olthoff et al.^6^ identified, through electron transmission spectroscopy (ETS) experiments on the CCl_2_Br_2_ molecule, two σ* shape resonances that lead to the dissociation of this system, resulting in Cl^–^ and Br^–^. These findings were corroborated by dissociative electron attachment (DEA) experiments, which identified two structures at 0.3 and 0.7 eV. The ETS results reported by Modelli et al.^7^ for the CH_2_Br_2_ molecule exhibit a σ* resonance signature at 1.93 eV. Confirmed by theoretical calculations using the discrete multiple scattering (MS-Xα) and continuous (CMS-Xα) methods, this resonance was estimated at 1.68 and 1.88 eV, respectively. Additionally, the MS-Xα calculation also estimated a low-energy structure at 0.20 eV. Zhao and Wang^8^ subsequently conducted low-energy electron elastic scattering calculations on CH_2_Br_2_ and CCl_2_Br_2_ molecules using the R-matrix method. These authors performed multiple calculations, aiming to describe the polarization effect, presenting four different levels of static-exchange plus polarization and a close-coupling (CC) calculation. Some levels of calculation exhibited three shape resonances (σ_1_^*^, σ_2_^*^, and π*) while others showed only two (σ_2_^*^ and π*). The authors reported on the sensitivity of the resonance positions with different calculations, noting that in calculations reporting two resonances, the low-lying resonance became a bound state.

In this study, integral, differential, and momentum-transfer elastic cross sections are presented for energies up to 20 eV. These cross sections were calculated using the Schwinger multichannel (SMC) method implemented with pseudopotentials and are obtained in the static-exchange and static-exchange plus polarization approximations. The main objective of the present study is to describe and characterize the resonance spectrum of these systems as well as to evaluate the effect of chlorination on the CH_2_Br_2_ molecule. Due to the substitution of a hydrogen atom with a chlorine atom, the resonance can stabilize, appearing at a lower energy, or destabilize, occurring at a higher energy relative to the resonance positions in the reference molecule.

This paper is organized as follows: Sections 2 and 3 present a brief description of the theoretical method along with the computational details employed in the bound state and scattering calculations. The results are presented and discussed in Section 4. Finally, a summary of our conclusions is provided in Section 5.

## 2 Theory

The elastic cross sections presented in this work were obtained using the SMC method^9,10^ implemented with the norm-conserving pseudopotentials proposed by Bachelet, Hamann, and Schlüter (BHS).^11^ These pseudopotentials were used to represent the nuclei and core electrons of the heavy atoms. The SMC method is a variational approach to calculating the scattering amplitude, accounting for significant effects that occur during electron–molecule collisions, such as exchange interactions, target polarization, and multichannel coupling. Since the SMC method has been reviewed in ref (12), here we will present only the aspects of this method that are pertinent to the present calculations. In the SMC method, the resulting expression for the scattering amplitude is as follows:
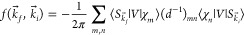
1where
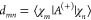
2and the operator *A*^(+)^ is given by
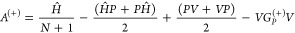
3

In the above equations, |*S*_*k⃗*_*i*(*f*)__⟩ is an eigenstate of the unperturbed Hamiltonian *H*_0_ = *H*_*N*_ + *T*_*N*+1_ and is given by the product of a target state and a plane wave with *k⃗*_*i*_(*f*) representing the momentum of the free incident (scattered) electron. In the definition of *H*_0_, *H*_*N*_ represents the target Hamiltonian and *T*_*N*+1_ corresponds to the kinetic energy operator of the incident electron. *V* is the interaction potential between the incident electron and the target’s electrons and nuclei; *Ĥ* = *E* – *H*, where *E* is the total collision energy and *H* is the (*N* + 1)-electron Hamiltonian in the fixed nuclei approximation; *P* is a projection operator onto the open-channel space of the target; and *G*_*P*_^(+)^ = *PG*_0_^(+)^ is the free-particle Green’s function projected into the *P*-space. |χ_*n*_⟩ represents a basis set of (*N* + 1)-electron Slater determinants (CSFs—configuration state functions), which are constructed as spin-adapted products of target states with single-particle scattering orbitals and are given in two approximations, namely, the static-exchange (SE) and the static-exchange plus polarization (SEP) approximations. In the SE approximation, the CFSs are constructed as
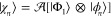
4where  is the antisymmetrization operator, |Φ_1_⟩ is the target ground state, and |ϕ_*n*_⟩ is a scattering orbital. In the SEP approximation, the configuration space used in the SE approximation is augmented by including CSFs constructed as
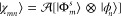
5where |Φ_*m*_^*s*^⟩ accounts for single virtual excitations of the target from the valence occupied (hole) orbitals of the ground (reference) state to a set of unoccupied (particle) orbitals with spin *s* (*s* = 0 for singlet or *s* = 1 for triplet states).

## 3 Computational Details

**Figure 1:**
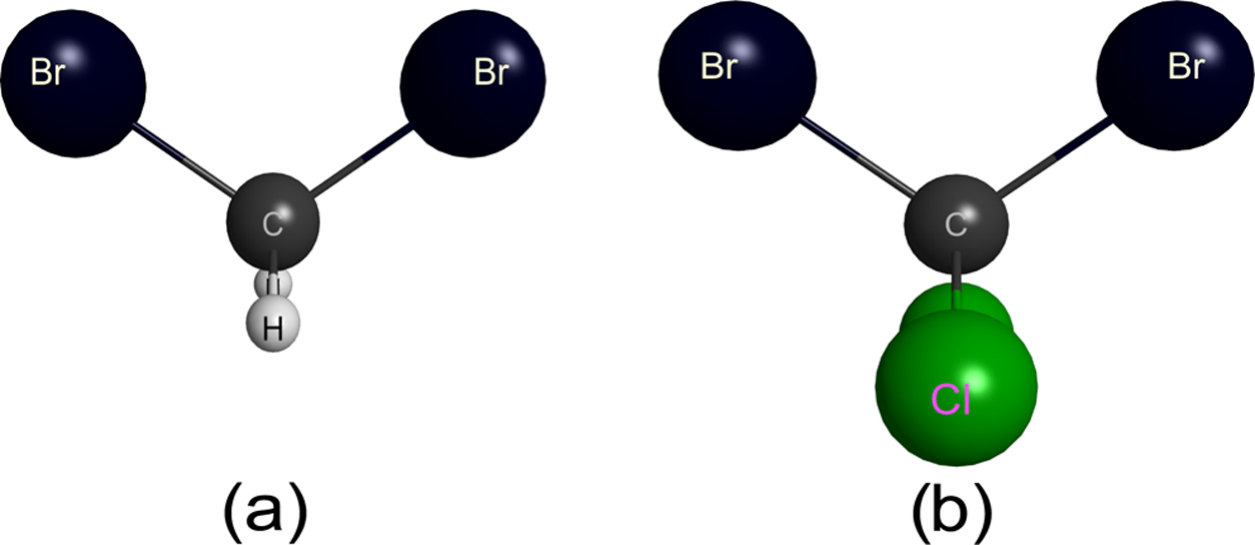
Ball and stick model of the (a) CH_2_Br_2_ and (b) CCl_2_Br_2_ molecules generated with MacMolPlt.^26^

The ground-state geometries of the molecular targets were optimized in the *C*_2*v*_ point group using second-order Møller–Plesset perturbation theory (MP2), with the aug-cc-pVDZ basis set, as implemented in the GAMESS package.^13^ The ball and stick models of both molecules are shown in Figure 1. In the SMC method, the ground state of the molecular target is described within the Hartree–Fock approximation. The valence electrons of the Br atom are represented by the 5s5p3d basis set, while those of the C and Cl atoms are represented by the 5s5p2d basis set. These basis sets are composed of Cartesian Gaussian (CG) functions generated as described by Bettega et al.^14^ The exponents of the CG functions are given in Table 1. For the hydrogen atoms, we used the 4s/3s basis set generated by Dunning Jr.,^15^ supplemented by one additional p-type function with exponent equal to 0.75.

**Table 1:** Exponents of the Uncontracted Cartesian Gaussian Functions Used for Carbon (C), Bromine (Br), and Chlorine (Cl) Atoms in the Present Calculations Performed with the SMC Method

type	C	Br	Cl
s	12.49628	7.743009	10.49065
s	2.470286	1.588291	6.836599
s	0.614028	0.385169	2.420592
s	0.184028	0.118779	0.513579
s	0.039982	0.026184	0.188863
p	5.228869	4.789276	6.037205
p	1.592058	1.856547	2.012401
p	0.568612	0.664700	0.686842
p	0.210326	0.265909	0.218056
p	0.072250	0.098552	0.071193
d	0.603592	0.233204	1.611766
d	0.156753	0.090186	0.328314
d		0.616528	

In the SEP approximation, we used modified virtual orbitals (MVOs),^16^ obtained from a cationic Fock operator with charge +6, to describe the particle and the scattering orbitals. To construct the configuration space used at this level, we adopted a strategy based on an energy cutoff following the relation ϵ_part_ – ϵ_hole_ + ϵ_scat_ < Δ,^17^ where ϵ_part_ is the particle orbital energy, ϵ_hole_ is the hole orbital energy, ϵ_scat_ is the scattering orbital energy, and Δ is the energy cutoff, for which we used the value of 2.0 hartree. For both molecules, we employed the same cutoff value, Δ, and considered singlet and triplet excitations for all symmetries. The number of CSFs generated according to the adopted criteria is presented in Table 2 for the two molecules considered in this work.

**Table 2:** Number of CSFs (*N*_CSF_) per Symmetry for the CH_2_Br_2_ and CCl_2_Br_2_ Molecules Used in the Scattering Calculations Carried Out at the SEP Level of Approximation

symmetry	CH_2_Br_2_	CCl_2_Br_2_
*A*_1_	4328	11 741
*A*_2_	4007	11 249
*B*_1_	4032	11 415
*B*_2_	4296	11 570

Both CH_2_Br_2_ and CCl_2_Br_2_ molecules are polar with dipole moment calculated values of 1.70 D (in good agreement with the experimental value of 1.43 D^18^) for CH_2_Br_2_ and 0.16 D for CCl_2_Br_2_. The molecular permanent dipole potential leads to a long-range interaction between the incident electron and the dipole potential. Consequently, we employed the Born-closure procedure to accurately incorporate the contribution of the higher partial waves. The Born-closure procedure combines the scattering amplitude obtained by the SMC method with the scattering amplitude of the dipole potential calculated in the first Born approximation (FBA). This method aims to enhance the accuracy of differential cross sections (DCSs) at low scattering angles. In essence, we expand the scattering amplitude obtained by the SMC method in partial waves up to a certain value of *l*_SMC_, as described in ref (12). For the scattering amplitude of the dipole potential, calculated in the FBA, the partial wave expansion is performed from (*l*_SMC_ + 1) to ∞. The *l*_SMC_ values are chosen by comparing the DCSs obtained with and without Born-closure, ensuring that they match above approximately 20°. The choice for the value of *l*_SMC_ depends on the energy of the incident electron, so in the present calculations, we choose for the CH_2_Br_2_ molecule: *l* = 1 for the interval from 0.1 to 0.5 eV, *l* = 2 from 0.6 to 2.0 eV, *l* = 3 from 2.1 to 3 eV, *l* = 4 from 3.1 to 3.4 eV, *l* = 5 from 3.5 to 4.0 eV, *l* = 6 from 4.1 to 6.5 eV, *l* = 7 from 6.6 to 7.0 eV, *l* = 8 from 7.1 to 8.5 eV, *l* = 9 from 8.6 to 9.5 eV and *l* = 10 from 9.6 to 50 eV. For the CCl_2_Br_2_ molecule, the *l* chosen were: *l* = 1 for the interval from 0.1 to 0.2 eV, *l* = 2 to 0.3 eV, *l* = 3 from 0.4 to 1.0 eV, *l* = 4 from 1.1 to 2.0 eV, *l* = 5 from 2.1 to 3.0 eV, *l* = 6 from 3.1 to 4.5 eV, *l* = 7 from 4.6 to 7.5 eV, *l* = 8 from 7.6 to 8.5 eV, *l* = 9 from 8.6 to 9.5 eV and *l* = 10 from 9.6 to 50 eV.

In order to assess and characterize the resonant structures observed in the elastic scattering cross sections, we proceeded to diagonalize the scattering Hamiltonian (*H*_*N*+1_) in the CSF space of the resonant symmetry. This diagonalization enables the search for resonant states of the (*N* + 1)-electron system. From the eigenvalues of *H*_*N*+1_ that lie close to the energy range of the resonances, it is possible to construct the Dyson orbitals |ϕ_*j*_⟩, as follows:
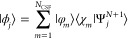
6where the sum runs over all of the *N*_CSF_ SE CSFs, |φ_*m*_⟩ is the scattering orbital used in the construction of |χ_*m*_⟩, and |Ψ_*j*_^*N*+1^⟩ represents the eigenvector of *H*_*N*+1_. These orbitals accurately represent the resonant states of the molecules.

## 4 Results and Discussion

The presentation of our results for low-energy elastic electron scattering by the CH_2_Br_2_ and CCl_2_Br_2_ molecules will be conducted as follows: first, we will present the scattering results for each molecule separately, discussing their characteristics and comparing them with the results currently available in the literature. Next, we will discuss the impact of replacing the H atoms with Cl atoms and highlight how this substitution affects the position of the resonances.

### 4.1 Dibromomethane

**Figure 2:**
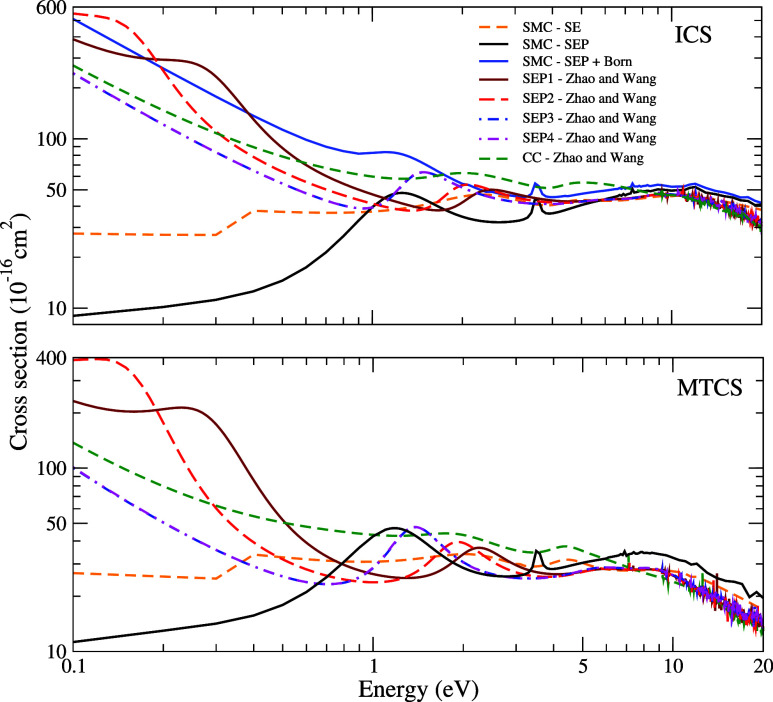
Cross sections for the elastic scattering of electrons by CH_2_Br_2_. Top: integral cross sections; bottom: momentum-transfer cross sections. Dashed orange line, SMC – SE results; full black line, SMC – SEP results; full sky blue line, SMC – SEP + Born results. The other results are obtained by Zhao and Wang^8^ using the R-matrix method in approximations: full vermillion line, SEP1; dashed red line, SEP2; dashed blue line, SEP3; double dotted-dashed magenta line, SEP4; dashed dark green line, CC. See the text for further discussion.

Our ICS (top panel) and MTCS (bottom panel) for electron scattering by the CH_2_Br_2_ molecule obtained in the SE, SEP, and SEP+Born approximations at impact energies from 0.1 to 20 eV are presented in Figure 2. In the SE approximation, we observe three structures centered at 2.20, 5.00, and 8.70 eV. These structures are shifted to lower energies in the SEP approximation, where the second and third structures are found at 1.24, and 5.60 eV. The first structure becomes a bound state. The structure centered at 3.50 eV which is nonphysical, appears due to linear dependency in the basis set of functions used in the construction of the CSF space. For energies above 7 eV, there are some pseudoresonances that originate from channels that are energetically accessible to the molecular target but are treated as closed at the SEP (1-channel) calculation level. The cross section obtained with the Born-closure procedure is consistent with previous results, indicating that using this procedure leads to an increase in the magnitude of the ICS at very low energies, but with no changes in the position of resonant or pseudoresonances. Also included in this figure are the results obtained by Zhao and Wang,^8^ who used the R-matrix method. In their work, these authors presented electron scattering calculations using four distinct levels of polarization in the SEP approximation along with a calculation carried out in the CC level of approximation. In the discussion that follows, we have maintained the same terminology used by the authors to index the different levels of calculation. The two structures identified in our calculations are also found in the SEP3 and SEP4 calculations performed by Zhao and Wang.^8^ The low-lying structure observed in our SE calculation becomes a bound state in the SEP3 and SEP4 calculations Zhao and Wang.^8^ From now on, we will focus the discussion on the results obtained with the SEP approximation due to the inclusion of polarization effects. For the first resonant structure, characterized as σ*-type resonance, the SEP1 and SEP2 results of Zhao and Wang^8^ locate the resonances at 0.27 and 0.14 eV respectively, while at the CC level, the resonance is centered at 2.11 eV. For the second resonance, characterized as a σ* type, our calculations place it at 1.24 eV. Zhao and Wang^8^ identified this resonance at 2.81 and 2.30 eV in the SEP1 and SEP2 levels, respectively, which are higher than the values reported by our calculations. For the SEP3 and SEP4 approximations, the same value of 1.61 eV was reported, which is closer to the value we obtained. In contrast, the authors’ CC calculation places the resonance significantly higher than our results, at 5.15 eV. Lastly, for the third resonance, characterized as a σ* type, our calculation places it at 5.60 eV, which is lower than the results obtained by Zhao and Wang^8^ of 7.36, 7.32, 6.74, and 6.74 eV for SEP1, SEP2, SEP3, and SEP4, respectively. The difference in the resonance position ranges from 1.14 to 1.76 eV. Overall, the cross sections exhibit similar behavior in terms of magnitude, while the differences in the present resonance positions compared to the results obtained using the R-matrix method are probably due to the different ways through which the polarization effects are treated in the two methods. Modelli et al.,^7^ in a combined theoretical and experimental study, identified two σ* resonances using the continuous multiple scattering Xα (CMS-Xα) and bound (MS-Xα) methods, and ETS. The ETS suggests a single structure (σ_2_^*^) centered at 1.93 eV, which is in good agreement with the results obtained in our calculations. The multiple scattering calculation estimates the position of this resonance at 1.68 eV, and the continuous multiple scattering CMS-Xα calculation estimates it at 1.88 eV, which is also in good agreement with our results. Only the multiple scattering MS-Xα calculation estimates the position of the first resonance (σ_1_^*^) at 0.20 eV. This low-lying σ* resonance was not observed in the ETS measurements, possibly due to the resolution of the apparatus. The authors investigated the resonant states of the CH_3_I molecule and its derivatives (CH_2_I_2_, CHI_3_, and CI_4_), finding that resonances in these derived molecules occur at very low energies, close to or below zero (bound state), compared to those in the CH_3_I system. This study provided the basis for the results obtained for bromomethanes derived from CH_3_Br (CH_2_Br_2_, CHBr_3_, and CBr_4_), where the resonant state becomes a bound state for these derived systems. Modelli et al.^7^ also measured the Br^–^ fragment using the dissociative attachment (DA) spectrum of the CH_2_Br_2_ molecule, identifying a sharp peak near 0.01 eV, corroborating the results presented by the authors. Guerra et al.,^19^ in their study involving the CH_3_Cl molecule and its derivatives (CH_2_Cl_2_, CHCl_3_, and CCl_4_) also identified a similar effect, where the resonant states become bound states compared to the CH_3_Cl molecule. The third resonance predicted in our calculations was not identified by the authors, possibly because this structure is very broad and, consequently, has a very short lifetime, making it difficult to detect, as discussed by the authors for fluoromethanes. The values of the resonance positions obtained in our study compared with the results found in the literature are summarized in Table 3.

**Table 3:** Comparison between the Positions of the Resonances (in eV) Observed in the Elastic Electron Scattering Obtained by the SMC Method and the Results Found in the Literature for the CH_2_Br_2_ and CCl_2_Br_2_ Molecules

CH_2_Br_2_	SE	SEP (SMC)	*H*_*N* + 1_	SEP1^8^	SEP2^8^	SEP3^8^	SEP4^8^	CC^8^	Ms-Xα^7^	ETS^7^	VAEs^25^
σ_1_^*^ (*A*_1_)	2.20		–0.13	0.27	0.14			2.11	0.20		–0.47
σ_2_^*^ (*B*_2_)	5.00	1.24	2.21	2.81	2.30	1.61	1.61	5.15	1.68	1.93	1.26
σ_3_*(*B*_1_)	8.70	5.60	7.64	7.36	7.32	6.74	6.74	7.10			3.78
CCl_2_Br_2_										(6)	
σ_1_^*^ (*A*_1_)	1.26	0.40	0.47	0.88	0.63	0.32	0.32	1.22		0.40	1.00
σ_2_^*^ (*B*_2_)	2.80		–0.29	1.24	0.95	0.63	0.63	4.32		1.00	–0.23
σ_3_*(*B*_1_)	4.20	0.67	0.77	0.24	0.09			6.48			1.15

**Figure 3:**
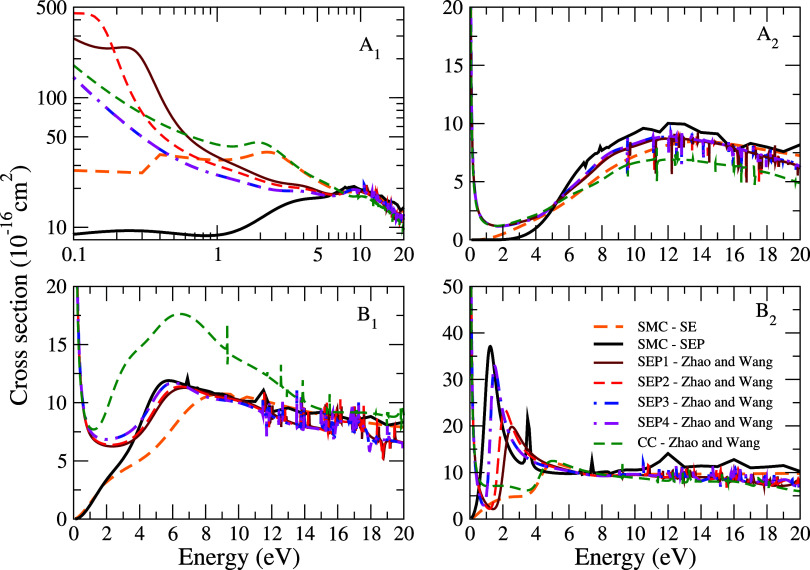
Symmetry decomposition of the integral cross section of CH_2_Br_2_ according to the *C*_2*v*_ point group. Dashed orange line, SMC – SE results; full black line, SMC – SEP results. The other results are obtained by Zhao and Wang^8^ using the R-matrix method in approximations: full vermillion line, SEP1; dashed red line, SEP2; dashed blue line, SEP3; double dotted-dashed magenta line, SEP4; dashed dark green line, CC. See the text for further discussion.

**Figure 4:**
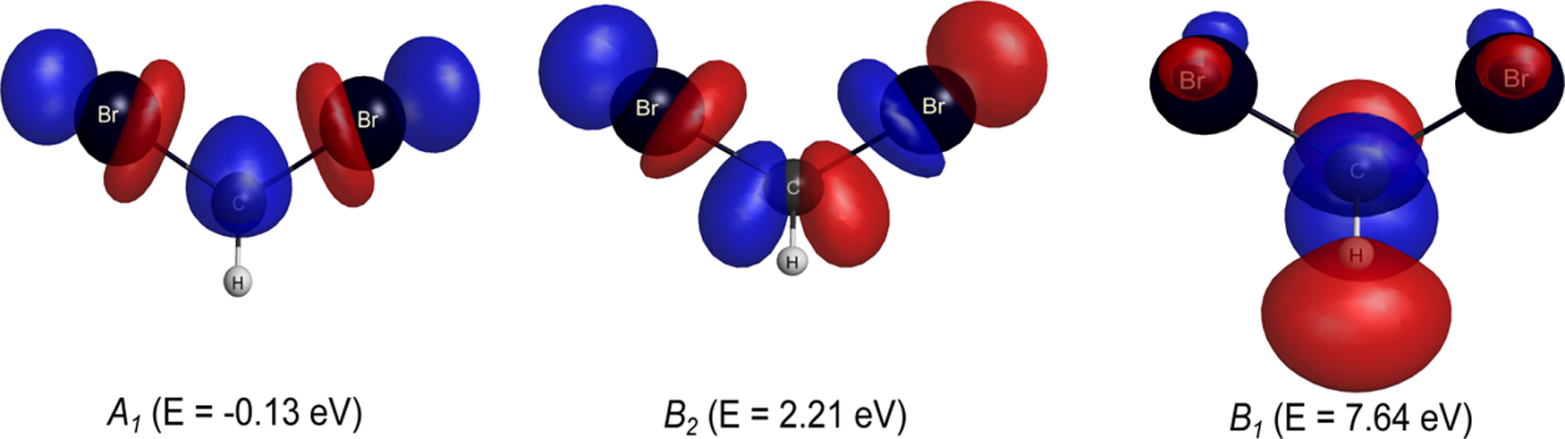
Resonant orbitals of CH_2_Br_2_. Left: σ_CBr_^*^ (*A*_1_); middle: σ_CBr_^*^ (*B*_2_); and right: σ_CH,CBr_^*^ (*B*_1_). See the text for further discussion.

In Figure 3, the symmetry decomposition of the ICS according to the *C*_2*v*_ point group for the CH_2_Br_2_ molecule, calculated in the SE and SEP approximations, is compared to the results obtained by means of the R-matrix method.^8^ The resonant structures observed in the ICS and MTCS originate from the *B*_2_ (first structure) and *B*_1_ (second structure) symmetries, in agreement with the results observed by Zhao and Wang.^8^ Although it does not present any visible signature in our cross-sectional curves, an anion-bound state was identified in the present results by the diagonalization of the scattering Hamiltonian, with a value of −0.13 eV in the *A*_1_ symmetry. The diagonalization of *H*_*N*+1_ also revealed two eigenstates in the *B*_2_ and *B*_1_ symmetries, with eigenvalues of 2.21 and 7.64 eV, energy values that are close to the region where the resonances were observed in our cross sections. In Figure 4, the Dyson orbitals obtained through the diagonalization of the scattering Hamiltonian for the eigenstates of the *A*_1_, *B*_2_, and *B*_1_ symmetries are shown. By inspection, we found that both for the bound state and for the second resonance, the orbitals are concentrated on the C–Br bond. Based on these results, it is possible to assign a σ* character with the second resonant state and with the bound state. On the other hand, for the third resonance, the orbital responsible for trapping the incident electron is strongly concentrated on the C–H bond, with a small contribution from the C–Br bond region. Accordingly, we assign a σ* character to this resonant state, which differs from the π* character attributed by Zhao and Wang.^8^ Despite these differences, in general, we observed excellent agreement in the magnitude and behavior of the cross sections compared to the R-matrix results for higher energies. Discrepancies observed at lower energies in the regime of low energies are attributed to the way polarization is treated in each method.

### 4.2 Dichlorodibromethane

**Figure 5:**
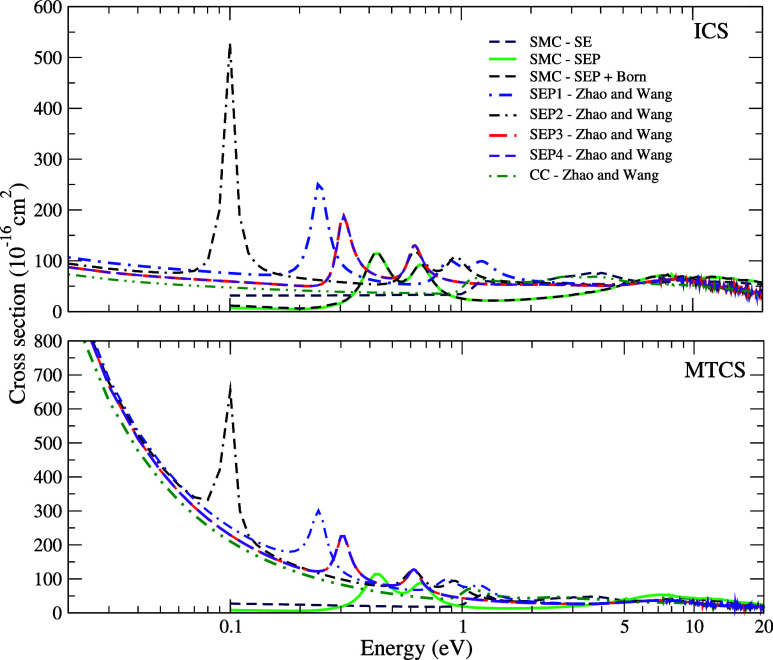
Cross sections for the elastic scattering of electrons by CCl_2_Br_2_. Top: integral cross sections; lower panel: momentum-transfer cross sections. Dashed royal blue line, SMC – SE results; full light green line, SMC – SEP results; dashed navy blue line, SMC – SEP + Born results. The other results are obtained by Zhao and Wang^8^ using the R-matrix method in approximations: dotted-dashed blue line, SEP1; double dashed-dotted black line, SEP2; long dashed red line, SEP3; short dashed violet line, SEP4; double dotted-dashed dark green line, CC. See the text for further discussion.

The ICS (top panel) and MTCS (bottom panel) for electron scattering by the CCl_2_Br_2_ molecule, calculated according to the SE, SEP, and SEP+Born approximations, are presented in Figure 5. The results also include the data obtained by Zhao and Wang^8^ using the R-matrix method. In the SE approximation, three pronounced structures are observed in the cross sections, which are centered at 1.26, 2.80, and 4.20 eV. When polarization effects are included in the SEP approximation without and with the Born-closure procedure, these structures shift to lower energies, resulting in two distinct structures centered at 0.40 and 0.67 eV. The second structure observed in the SE approximation becomes a bound state when polarization is included. There are some structures present in the cross sections above 7 eV ascribed to pseudoresonances, as already discussed before. As for the CH_2_Br_2_ molecule, Zhao and Wang^8^ conducted different tests of polarization on the CCl_2_Br_2_ molecule. The authors presented four different calculations in the SEP approximation, along with a CC calculation, and once again, we kept the same nomenclature used by these authors. When comparing our results obtained in the SEP approximation with those obtained in the SEP1 approximation by Zhao and Wang,^8^ the first structure, characterized as σ* type, is centered at 0.88 eV, which is higher than the value we obtained. In the SEP2 approximation, which includes a larger number of virtual orbitals and active electrons, the resonance tends to be at a lower energy, more precisely, centered at 0.63 eV, but still above the value found in our work. However, in the SEP3 and SEP4 approximations, the center of the resonance is placed at 0.32 eV, showing good agreement with our results. In the CC model, the resonance is positioned at 1.22 eV, significantly above the value obtained in our work. The discrepancies in the positions of present resonances compared to the results obtained by Zhao and Wang^8^ can be attributed to the different number of CSFs considered in the description of polarization. Olthoff et al.^6^ conducted ETS measurements and observed two σ*-type resonances, around 0.4 and 1.0 eV. Additionally, through DEA, they measured the yield of negative fragments. The energies for Cl^–^ and Br^–^ ion production due to dissociative capture are close to 0.3 and 0.7 eV, respectively, in agreement with the results obtained by the use of the ETS technique. The first resonance observed in our calculations is in excellent agreement with the authors’ results; however, the second resonance becomes a bound state in our calculations. Table 3 summarizes the values obtained for the resonance positions along with the data found in the literature.

**Figure 6:**
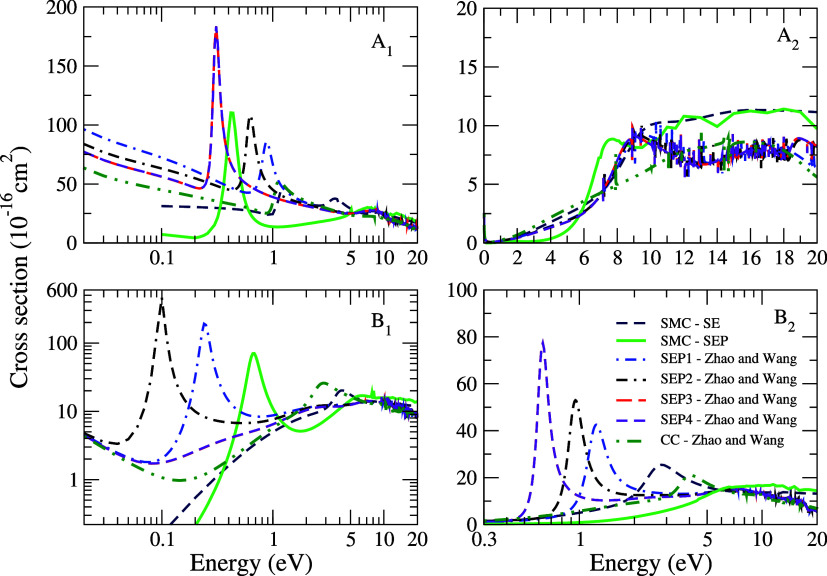
Symmetry decomposition of the integral cross section of CCl_2_Br_2_ according to the *C*_2*v*_ point group. Dashed royal blue line, SMC – SE results; full light green line, SMC – SEP results. The other results are obtained by Zhao and Wang^8^ using the R-matrix method in approximations: dotted-dashed blue line, SEP1; double dashed-dotted black line, SEP2; long dashed red line, SEP3; short dashed violet line, SEP4; double dotted-dashed dark green line, CC. See the text for further discussion.

**Figure 7:**
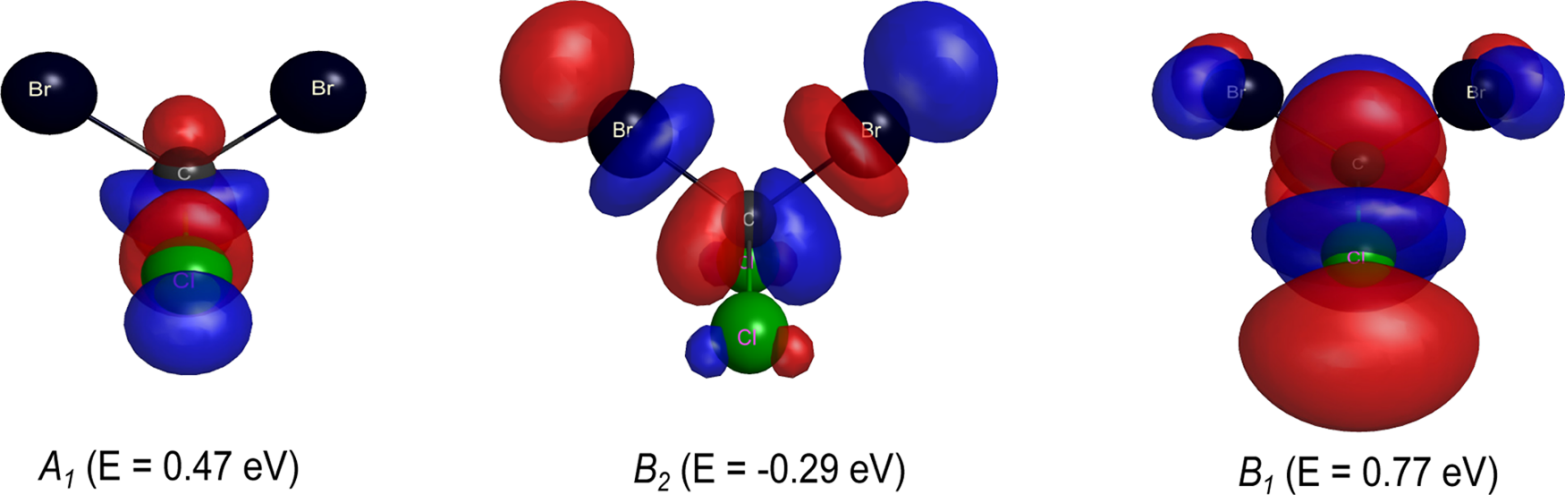
Resonant orbitals of CCl_2_Br_2_. Left: σ_CCl_^*^ (*A*_1_); middle: σ_CBr,CCl_^*^ (*B*_2_); and right: σ_CBr,CCl_^*^ (*B*_1_). See the text for further discussion.

In Figure 6, we present the symmetry decomposition of the ICS according to the *C*_2*v*_ point group, calculated in the SE and SEP approximations for electron scattering by the CCl_2_Br_2_ molecule. The resonant structures observed in the ICS originate from the *A*_1_, *B*_2_, and *B*_1_ symmetries, which agrees with the results obtained by Zhao and Wang.^8^ It is noted that when considering polarization effects in the SEP approximation, the second resonance present in the *B*_2_ symmetry becomes a bound state and therefore shows no signature in the cross section. The diagonalization of the scattering Hamiltonian indicates the presence of eigenstates in the *A*_1_, *B*_2_, and *B*_1_ symmetries with eigenvalues of 0.47, −0.29, and 0.77 eV, respectively. The eigenvalues for the *A*_1_ and *B*_1_ symmetries agree well with the positions of the resonances obtained from our scattering calculations. As previously mentioned, when considering polarization effects on the resonance present in the *B*_2_ symmetry, it becomes a bound state, which corroborates the value obtained from the diagonalization of the scattering Hamiltonian. According to the orbitals obtained through diagonalization, the lowest energy resonance is concentrated in the C–Cl bond region, as shown in Figure 7. The second resonance, which becomes a bound state, is concentrated in the C–Br bond region, with a small contribution from the Br–Cl bond. The third resonance is concentrated along the C–Cl bond, with a contribution from the C–Br bond as well. In general, the cross sections obtained in this study agree well in terms of behavior and magnitude with the results of Zhao and Wang.^8^ The differences at lower energies are attributed to the different treatments of polarization in each method.

### 4.3 Chlorination Effects

In this subsection, our focus will be to present and discuss the differences in the resonance positions that appear in the ICS of the CH_2_Br_2_ and CCl_2_Br_2_ molecules. Several studies have addressed the issue of how the substitution or addition of a heteroatom^17,20−22^ or functional group^23,24^ in a reference molecular structure affects the position of a resonance. These studies indicate that such modifications can stabilize or destabilize the resonance, i.e., shifting it to lower or higher energies, depending on the effect of the atom or functional group.

**Figure 8:**
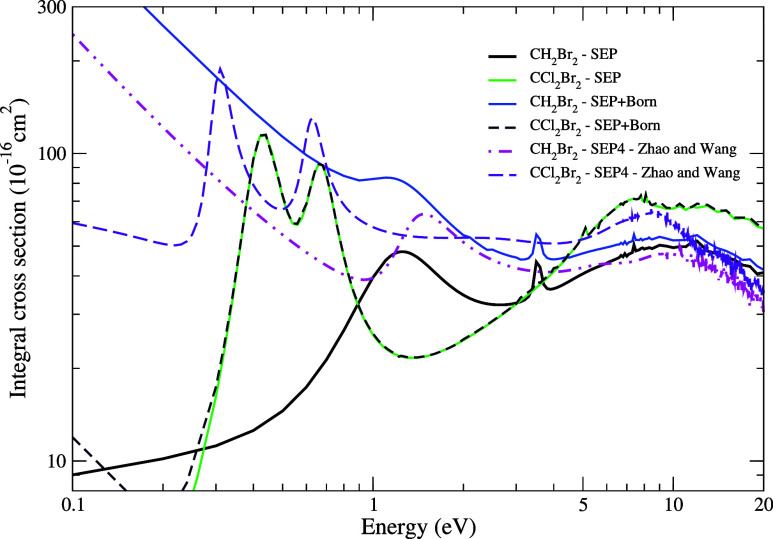
Integral cross sections for the elastic scattering of electrons by CH_2_Br_2_ and CCl_2_Br_2_ molecules. For CH_2_Br_2_: Full black line, SMC – SEP results; full sky blue line, SMC – SEP + Born results; and double dotted-dashed magenta line, SEP4 obtained by Zhao and Wang.^8^ For CCl_2_Br_2_: Full light green line, SMC – SEP results; dashed navy blue line, SMC – SEP + Born results; and short dashed violet line, SEP4 obtained by Zhao and Wang.^8^ See the text for further discussion.

In Figure 8, we present the ICS for elastic electron scattering by the CH_2_Br_2_ and CCl_2_Br_2_ molecules using the SEP approximation both with and without the Born-closure procedure. Additionally, we compare our results with those obtained by Zhao and Wang^8^ using the SEP4 calculation which, as emphasized by the authors, it is the level of calculation that provides a better description of polarization effects at lower energies. In general, replacing the H atom with a Cl atom in the molecule results in an ICS of greater magnitude. At low energies, there is a sharp increase in the cross section due to the significantly larger permanent dipole moment of the CH_2_Br_2_ molecule compared to that for the CCl_2_Br_2_ molecule, more precisely, a difference of 1.54 D. This effect is evident when considering the long-range dipole potential correction in the SEP+Born approximation.

Regarding the resonance positions, chlorination of the molecule leads to destabilization of the first resonance. In the CH_2_Br_2_ molecule, the resonance is concentrated in the C–Br bond region, while in the CCl_2_Br_2_ molecule, it moves to the C–Cl bond. The bond lengths for C–Br and C–Cl are 1.9370 and 1.7646 Å, respectively. This suggests that longer bond lengths result in a more positive region, making it more attractive to the continuum electron. The substitution of the hydrogen atom by a chlorine atom tends to stabilize the second and third resonances. In the second resonance, the concentration is in the C–Br bond region for both systems, with a small contribution from the C–Cl bond. The C–Br bond length is 1.9565 Å in the CCl_2_Br_2_ molecule, which is longer than in the CH_2_Br_2_ molecule, indicating that the C–Br region is more positive in the CCl_2_Br_2_ molecule and consequently more attractive to the incoming electron. Additionally, the net charge on the C–Br bond is −0.4892 in the CH_2_Br_2_ molecule and −0.1824 in the CCl_2_Br_2_ molecule. This outcome suggests that substituting hydrogen with chlorine stabilizes the resonance, highlighting the chlorination effect. The inductive effect of the chlorine atom plays a significant role due to its higher electronegativity compared with the carbon atom. The inductive effect refers to the ability of an atom or group of atoms to influence the distribution of charge along a bond based on differences in electronegativity. In this case, chlorine, being more electronegative, pulls electron density toward itself, creating a charge imbalance that affects adjacent bonds. For the third resonance, the orbitals concentrate around the C–H and C–Cl bonds in the CH_2_Br_2_ and CCl_2_Br_2_ molecules, respectively, with a small contribution from the C–Br bond in both systems. The C–H bond length is 1.0859 Å, which is shorter than the C–Cl bond length. Thus, the resonance tends to stabilize in the CCl_2_Br_2_ molecule, as indicated by our results. Through electronic structure calculations, it is possible to use the energy of the virtual orbitals to estimate the vertical attachment energies (VAEs) based on Koopmans’ theorem and with the aid of an empirical scaling relation.^25^ The 6-31G(d) basis set was employed to optimize the geometry of the molecule at the MP2 level, followed by a Hartree–Fock calculation using the same basis set. The resonance structure estimates for the molecules CH_2_Br_2_ and CCl_2_Br_2_ were as follows: for CH_2_Br_2_, σ_2_^*^ was estimated at 1.26 eV, σ_3_^*^ at 3.78 eV, and a bound state at σ_1_^*^ at −0.47 eV; for CCl_2_Br_2_, σ_1_^*^ was 1.00 eV, σ_3_^*^ was 1.15 eV, and a bound state at σ_2_^*^ at −0.23 eV. The obtained VAE values are summarized in Table 3. These estimates support the stabilization and destabilization effects of the resonances observed in the scattering calculations. Our results are in good agreement with what was observed by Zhao and Wang,^8^ and consistent with the experimental results obtained by Modelli et al.^7^ and Olthoff et al.^6^ for the second resonance.

**Figure 9:**
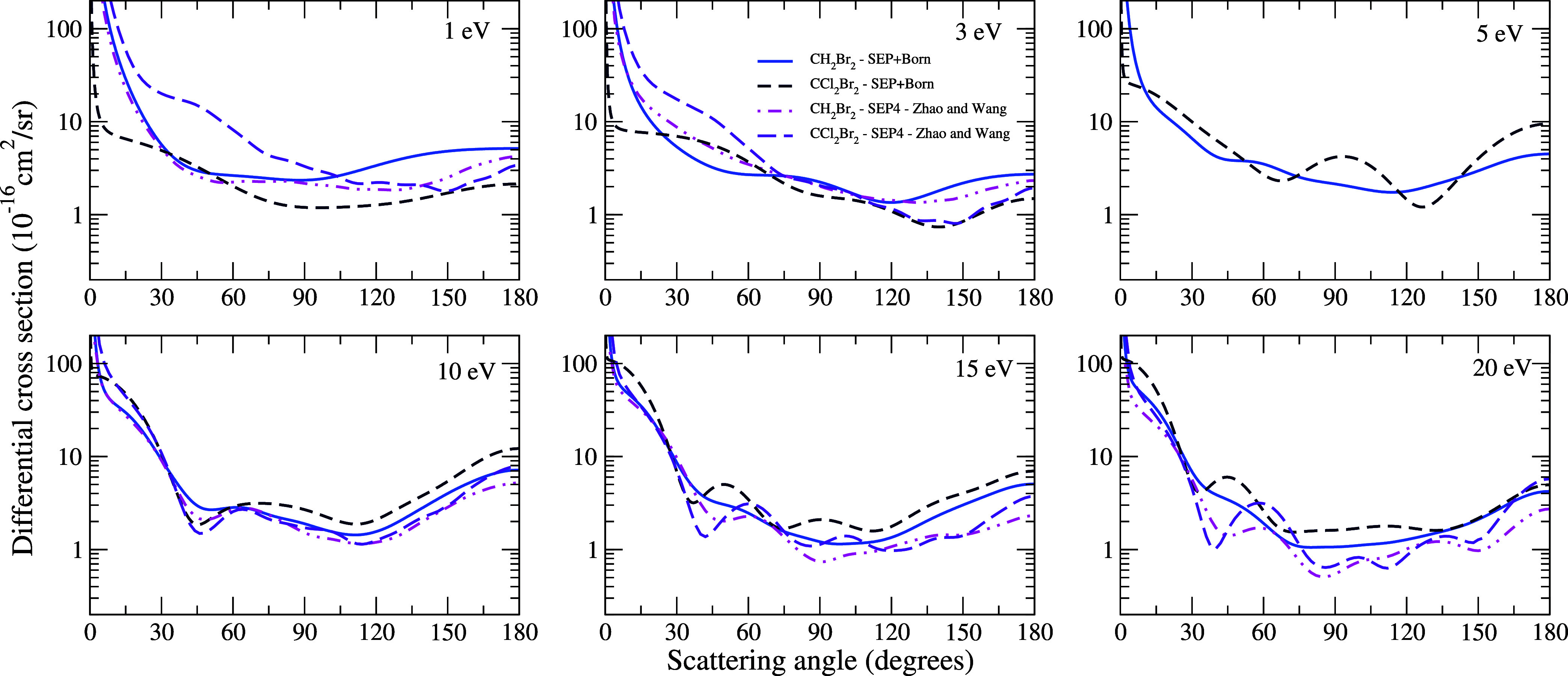
Differential cross sections for the scattering of electrons by CH_2_Br_2_ and CCl_2_Br_2_ molecules for impact energies of 1, 3, 5, 10, 15, and 20 eV. For CH_2_Br_2_: Full sky blue line, SMC – SEP+Born results; and double dotted-dashed magenta line, SEP4 obtained by Zhao and Wang.^8^ For CCl_2_Br_2_: Dashed navy blue line, SMC – SEP+Born results; and short dashed violet line, SEP4 obtained by Zhao and Wang.^8^ See the text for further discussion.

In Figure 9, we present the DCSs for elastic electron scattering by the CH_2_Br_2_ and CCl_2_Br_2_ molecules at the impact energies of 1, 3, 5, 10, 15, and 20 eV, calculated using the SEP + Born approximation, along with those obtained by Zhao and Wang.^8^ For the energy of 1 eV, the effect of the long-range dipole potential dominates the cross section due to the significant difference in the dipole values between the two systems. At 3 eV, an oscillatory pattern begins to be seen, with the occurrence of two slight minima at 60° and 120° for the CH_2_Br_2_ molecule, indicating a d-wave pattern. Similarly, for the CCl_2_Br_2_ molecule, two minima are observed at 90° and 135°. For this system, we find good agreement with Zhao and Wang’s results above 105°. At 5 eV, the minima positioned around 60° and 120° become more pronounced for the CCl_2_Br_2_ molecule, again indicating a *d*-wave pattern. At 10 eV, both systems show two minima, further confirming the d-wave pattern and showing excellent agreement with Zhao and Wang’s results. At 15 eV, three minima start to be discerned in the DCS for the CCl_2_Br_2_ molecule, indicating a dominant f-wave pattern. The differences in DCSs at 15 and 20 eV can be attributed to the number of structures observed in the ICS, due to pseudoresonances. Overall, substituting a hydrogen atom with a chlorine atom results in a more pronounced oscillatory pattern.

## 5 Conclusions

We presented the ICS, MTCS, and DCSs for elastic electron scattering by the CH_2_Br_2_ and CCl_2_Br_2_ molecules, calculated using the SMC method for impact energies up to 20 eV. Our results were compared with the theoretical data available to date, which were obtained by Zhao and Wang^8^ using the R-matrix method. We found that our results are in good agreement compared to the ones reported by these authors, with the differences in the resonance positions attributed to the different strategies used to describe polarization effects in both methods. The resonance positions identified by Modelli et al.^7^ and Olthoff et al.,^6^ through ETS experiments and multiple scattering^7^ calculations, also displayed good agreement with our assignments.

For the CH_2_Br_2_ molecule, we identified two shape resonances, centered at 1.24 eV (σ_CBr_^*^, *B*_2_) and 5.60 eV (σ_CH, Br_^*^, *B*_1_), respectively, and also a bound state. The energy of the bound state, with a value of −0.13 eV (σ_CBr_^*^, *A*_1_), was obtained via the diagonalization of the scattering Hamiltonian. With the substitution of H atoms by Cl atoms, the resonances shifted to 0.40 eV (σ_CCl_^*^, *A*_1_) and 0.67 eV (σ_CBr, CCl_^*^, *B*_1_), and a bound state obtained through the diagonalization of the Hamiltonian is observed at −0.29 eV (σ_CBr, CCl_^*^, *B*_2_). Analysis in terms of bond length, net charge, inductive effect, and the position of the resonances/bound state estimated via empirical scaling relation corroborates the results observed through scattering calculations obtained by the SMC method. In the low-energy regime, the change in the position of the resonances was the most significant difference observed, while at higher impact energies, the influence of the chlorination effect was expressed as a difference in the magnitude of the cross sections, as expected.

In this work, our objective was to complement the existing literature^6−8^ with a detailed analysis of the chlorination effect through the study of elastic electron scattering by the CH_2_Br_2_ and CCl_2_Br_2_ molecules, using fully ab initio calculations. With these results, we aim to encourage further theoretical and experimental research on electron scattering involving these systems in order to deepen and expand our analyzes.
